# Electronic and Magnetic Properties of Manganese Bromide Monolayers

**DOI:** 10.1021/acs.langmuir.4c04499

**Published:** 2025-02-25

**Authors:** Guilherme Carlos Carvalho de Jesus, Railson da Conceição Vasconcelos, Lucas Bezerra do Vale, Rafael Ferreira de Menezes, Kayla G. Sprenger, Ricardo Gargano

**Affiliations:** † Institute of Physics, University of Brasília, Campus Darcy Ribeiro, Brasília, DF 70910-900, Brazil; ‡ Department of Chemical and Biological Engineering, University of Colorado Boulder, Boulder, Colorado 80309-0401, United States

## Abstract

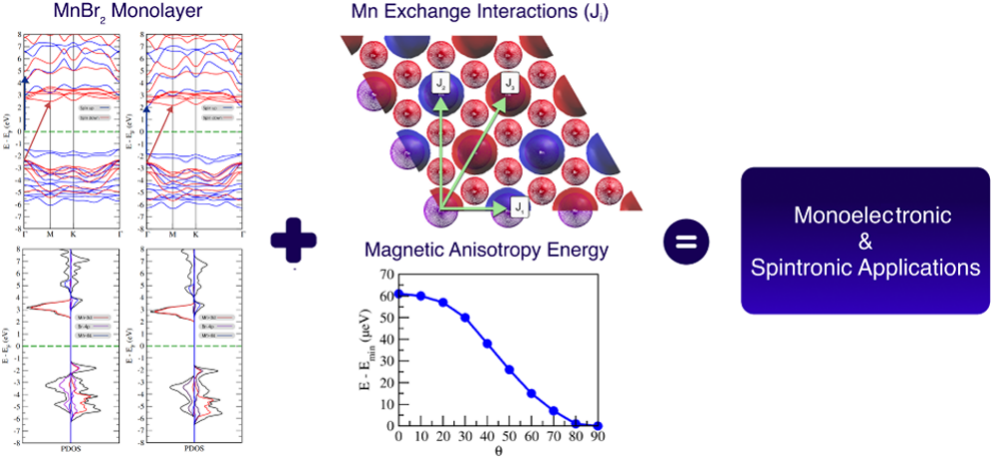

This study investigates the electronic and magnetic properties of monolayer and bulk manganese bromide (MnBr_2_) through *ab initio* simulations based on density functional. We computed the lattice parameters, band structures, and projected density of states, shedding light on the intrinsic magnetic behavior of MnBr_2_. Our analysis of atomic magnetic moments indicates that the incomplete 3d orbital of the manganese atom, containing five electrons, drives the material’s intrinsic magnetism. Additionally, our simulations reveal that the antiferromagnetic configuration is energetically more stable than the ferromagnetic configuration. Notable, We find that the MnBr_2_ system achieves its lowest energy state when the manganese magnetic moments are aligned perpendicular to the monolayer supercell plane. These findings highlight manganese bromide’s potential as a candidate for future applications in nanoelectronics and spintronics.

## 1 Introduction

The study of two-dimensional (2D) materials has experienced rapid growth in recent years due to their unique properties and broad technological applications.^1−5^ These ultrathin structures offer immense potential in fields such as electronics, energy storage, and optoelectronics. Their tunable properties have captured the interest of researchers worldwide, driving substantial advances in nanotechnology and paving the way for innovations like high-speed transistors and flexible sensors.^6,7^ As a result, 2D materials have become a focal point of cutting-edge research, presenting exciting possibilities for future technologies.^6,7^

Transition metal dichalcogenides (TMD), such as molybdenum disulfide (MoS_2_) and tungsten disulfide (WS_2_), exhibit intriguing physical properties. Detailed studies on charge transport and photoluminescence in TMD monolayers have been widely reported in the literature,^8−10^ revealing that while bulk TMDs exhibit an indirect band gap, their monolayer counterparts possess a direct band gap, making them more efficient at absorbing and emitting energy in the visible spectrum.^11^ Moreover, these materials exhibit excitonic states^12^ and unique electronic transport characteristics when synthesized as monolayers (grown by vapor decomposition^13^), as well as novel applications in optoelectronics when modified by strain.^14^ Graphene, another extensively studied 2D material, is composed of a single atomic layer and possesses several notable electronic properties. Graphene nanoribbons, in particular, exhibit varying electronic behaviors depending on their edge configuration and width: armchair-edge nanoribbons display semiconductor behavior, while zigzag-edge configurations exhibit semimetallic properties.^15−17^

More recently, manganese bromide (MnBr_2_) has attracted significant research interest due to its unique intrinsic properties. Studies indicate that MnBr_2_ behaves as a magnetic semiconductor, with its inherent magnetism stemming from the manganese atoms. Furthermore, the antiferromagnetic arrangement of the MnBr_2_ monolayer has been experimentally confirmed through neutron diffraction.^18^ From an application perspective, this material has been utilized in optical displays based on luminescence,^19,20^ the development of bioactive compounds,^21^ and the production of more efficient solar cells.^20,22,23^ Motivated by these promising features, this work investigates the electronic and magnetic properties of MnBr_2_ (in both bulk and monolayer forms) via computational simulations using *ab initio* methods, aiming to reveal further insights into expanding its potential technological applications.

## 2 Methodologies and Computational Details

The optimized geometric structure and total energy of MnBr_2_ were calculated using spin-polarized density functional theory (DFT) as implemented in the SIESTA^24^ computational package. The exchange-correlation functional was treated using the Perdew–Burke–Ernzerhof (PBE) approach within the generalized gradient approximation (GGA).^25^ Additionally, we employed Troullier–Martins pseudopotentials,^26^ a double-ζ polarized (DZP) basis set, a mesh cutoff of 400 Ry for the real-space grid, and an energy convergence criterion of 10^–5^ eV. The projected density of states (PDOS) was obtained by integrating the Brillouin zone with a 60 × 60 × 60 Monkhorst–Pack *k*-point mesh^27^ for the bulk form and 60 × 60 × 1 for the monolayer. The unit cell included a vacuum layer for the monolayer simulations to prevent self-interactions due to periodic boundary conditions. We used a 9 × 9 × 9 Γ-centered Monkhorst–Pack *k*-point mesh for the bulk form and 9 × 9 × 1 for the monolayer. The phonon band structure was obtained using a 3 × 3 × 1 supercell of the monolayer via the SIESTA computational package. From the results obtained, the postprocessing tool vibra (coupled in the SIESTA code) was used to determine the vibration frequencies of the lattice.

To estimate the magnetic isotropic exchange interactions in the Heisenberg model,^28^ we employed an alternative approach based on the Green’s function method, which uses the magnetic force theorem proposed by Liechtenstein, Katsnelson, Antropov, and Gubanov (LKAG).^29^ The LKAG method accurately captures magnetic properties without the computational burden often associated with energy and spin-spiral mapping methods,^30^ making it well-suited for high-throughput analyses. This methodology was implemented using the TB2J computational package,^31^ which processes the Hamiltonian output files from SIESTA. Calculations were performed using a 3 × 3 × 1 supercell for both monolayer and bulk forms, with highly accurate norm-conserving pseudopotentials incorporating full relativistic corrections, including spin–orbit coupling, sourced from the PseudoDojo database.^32^ For the bulk form, we used a 15 × 15 × 15 *k*-point mesh in reciprocal space, using DZP basis set, a mesh cutoff of 600 Ry for the real-space grid, and a total energy convergence criterion of 10^–5^ eV. A 21 × 21 × 1 *k*-point mesh was employed for the monolayer structure, along with the same basis set and energy convergence criterion, but with a mesh cutoff of 400.

## 3 Results and Discussion

### 3.1 Structural Properties of MnBr_2_ in Bulk and Monolayer Forms

**Figure 1:**
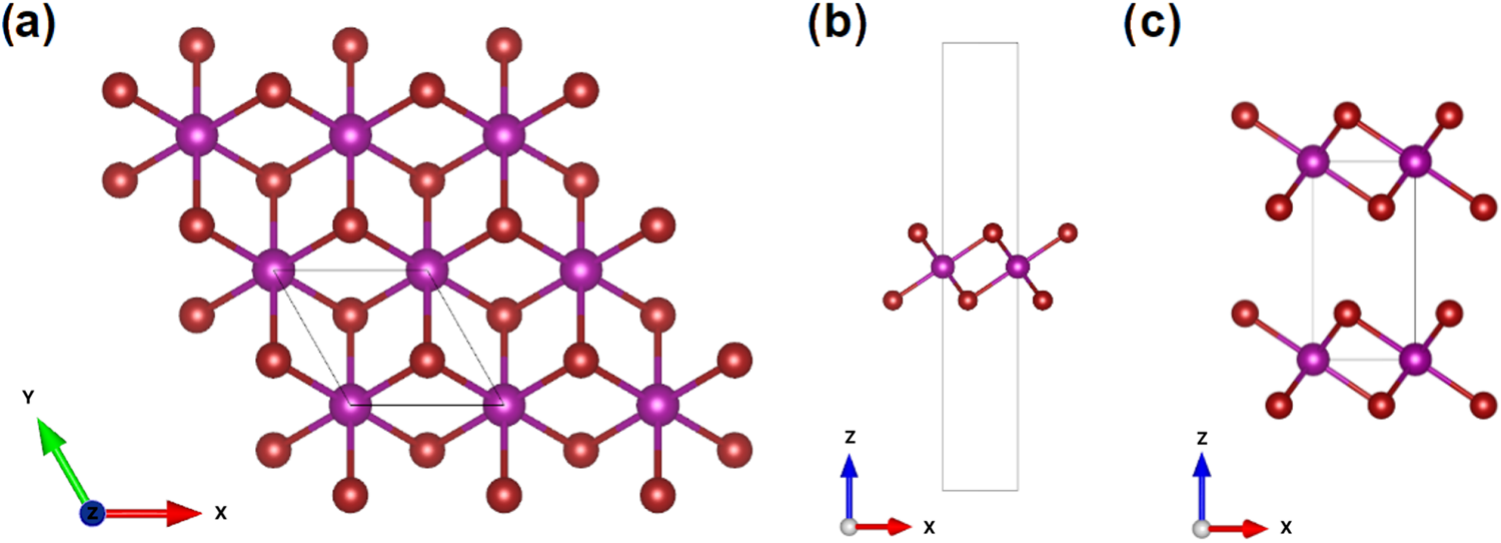
(a) Visualization of the MnBr_2_ monolayer from the *z*-axis, where the dashed line indicates the unit cell, (b) representation of the MnBr_2_ monolayer from the *y*-axis, and (c) visualization of the bulk form of MnBr_2_ around the *z*-axis. The purple and red colors indicate the Mn and Br atoms, respectively.

Figure 1 shows the simulated structures of MnBr_2_ in both bulk and monolayer forms from different perspectives. The simulations were based on established bulk parameters of MnBr_2_.^18^ In its bulk form, MnBr_2_ exhibits magnetic ordering and belongs to the space group P3 m1, characterized by a hexagonal arrangement. The lattice constants determined in this work were *a* = *b* = 3.87 Å, and *c* = 6.48 Å for the bulk form and *a* = *b* = 3.87 Å for the monolayer, consistent with the values reported in the literature.^18^ Furthermore, for the case where Mn and Br are located in the same monolayer, the result obtained for the distance between them is 2.72 Å, while for the distance between the Mn atoms it is 3.87 Å and they agree with those found in the literature.^33^ For the situation where Mn and Br are in different monolayers, the distance between them is 6.48 Å.

**Figure 2:**
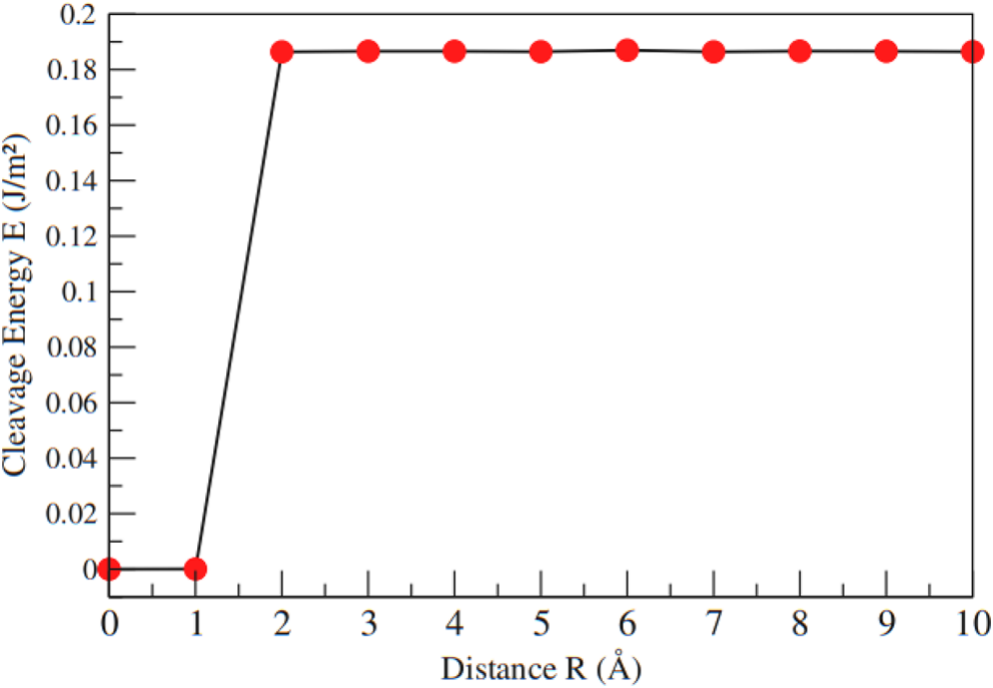
Energy as a function of the distance between the overlapping layers.

The transition from bulk to monolayer can be achieved using the well-known experimental cleavage technique. From a theoretical point of view, the cleavage energy was obtained by calculating the variation of the total energy (*E*) of the ground state concerning the separation distance between the two fracture parts (*R*) as shown in Figure 2. During this procedure, the lattice constants of a and b remain fixed with the values at the equilibrium state of MnBr_2_ in bulk form. Finally, the cleavage energy was determined by dividing the converged electron energy by the unit cell area, similar to the procedure described in ref^34^ In this study, cleavage was simulated by calculating *E* for varying *R* from 1 to 10 Å, with increments of 1 Å. The *E*(*R*) curve is shown in Figure 2. The obtained cleavage energy was 0.18 J/m^2^, which is lower than that of graphite cleavage energy^35^ (0.37 J/m^2^), indicating that MnBr_2_ monolayers could potentially be obtained from bulk material via mechanical microexfoliation.

**Figure 3:**
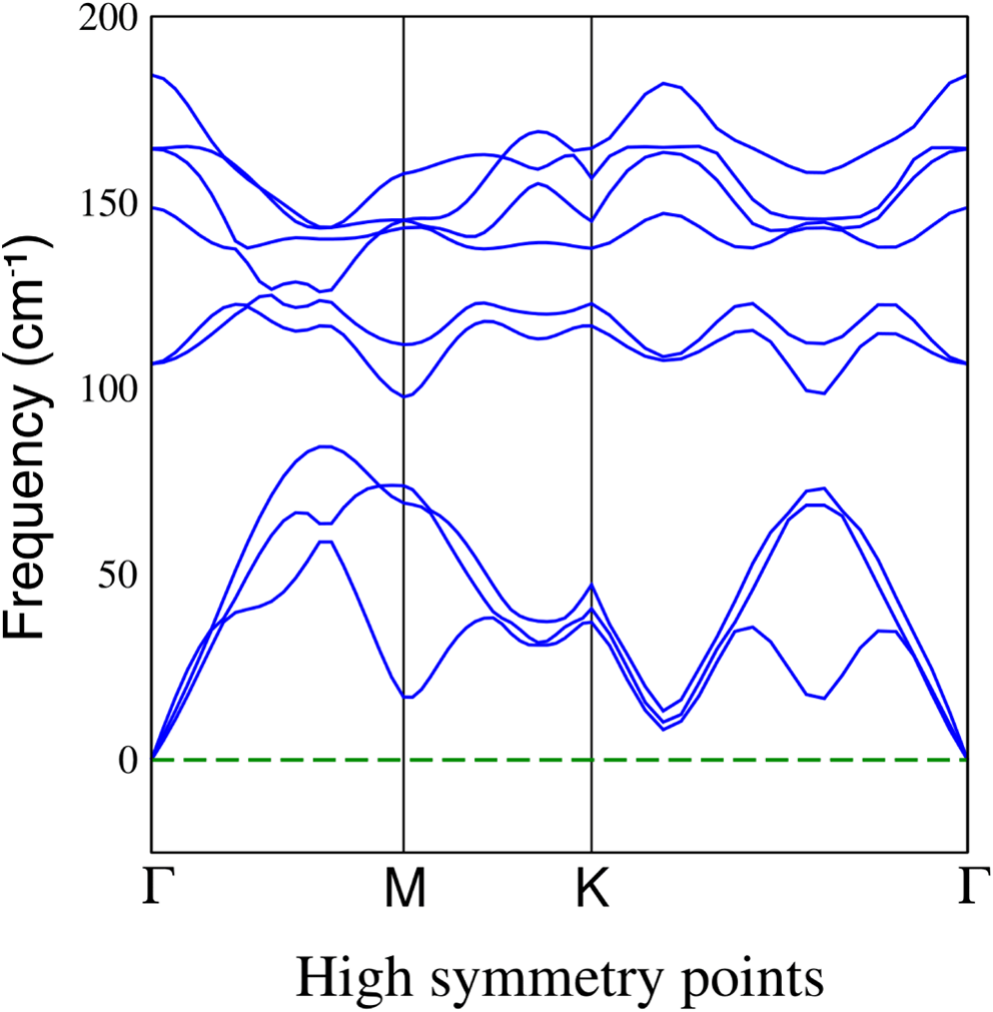
Phonon frequencies at some high-symmetry points on Brillouin zone for MnBr_2_ monolayer. The green dotted lines indicate the zero frequency level.

To better assess the mechanic properties of the MnBr_2_ monolayer, we determined the phonon frequencies at some high-symmetry points of this material (Figure 3). From Figure 3, it is possible to verify that the frequencies are all positive, showing no imaginary frequency modes, suggesting that the material can be considered dynamically stable.

### 3.2 Electronic and Magnetic Band Structures of MnBr_2_

**Figure 4:**
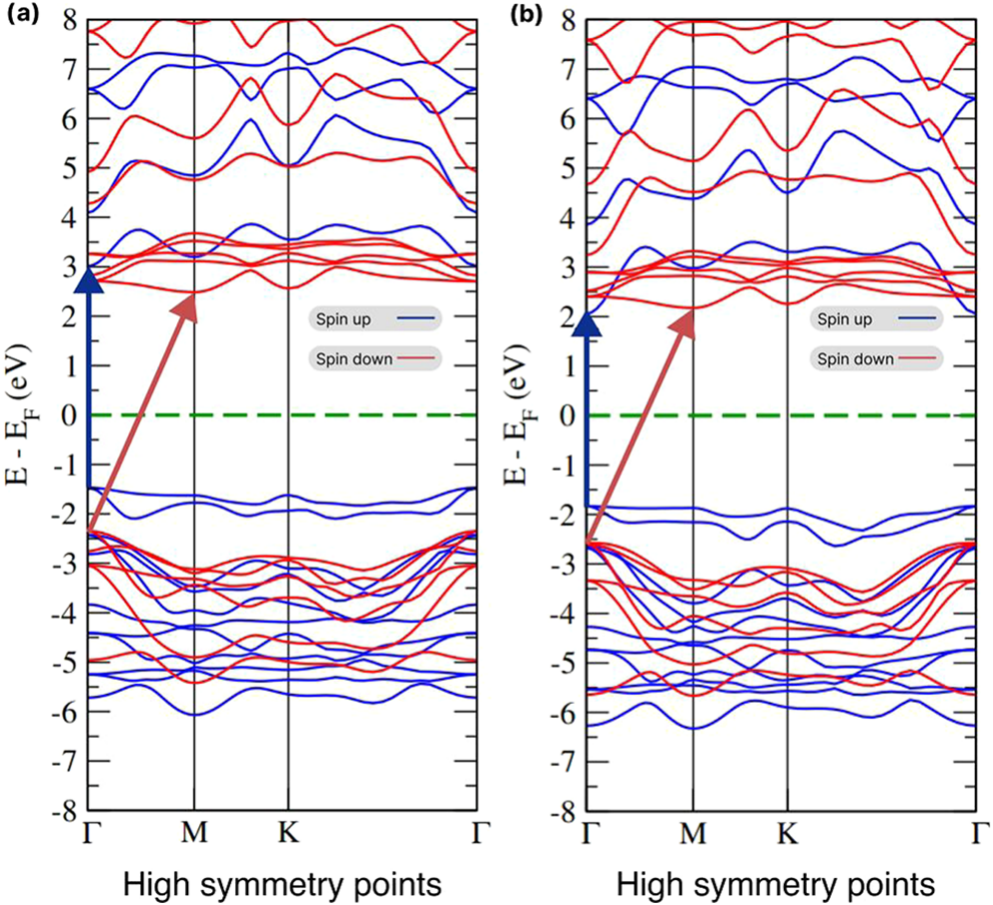
(a) Band structures of MnBr_2_ in bulk and (b) monolayer forms. The green dotted lines indicate the Fermi level (*E*_F_).

The electronic and magnetic properties of MnBr_2_ in both bulk and monolayer forms were analyzed through their respective band structures, shown in Figure 4. Both forms exhibit similar band structure patterns. In the bulk form, a direct band gap of 3.88 eV appears at the Γ point in the spin-up channel, while the spin-down channel shows an indirect band gap of 4.75 eV (Γ-M).

Knowing that the monolayers of the material in bulk form interact by van der Waals forces, the DFT-D3 method of Grimme et al.^36^ was used to correct the dispersion energy in the system. The obtained result showed that the difference between the energies with and without the DFT-D3 method for the Fermi level was 0.018 eV. For the total electronic energy, this difference was 0.59 eV. These results suggest that the use of the DFT-D3 method is not important to study this material.

In the monolayer form, the spin-up band gap remains direct (3.98 eV at Γ), while the spin-down channel displays an indirect band gap of 4.83 eV. Additionally, the monolayer shows an indirect gap of 3.95 eV, consistent with the literature, though with a slight energy shift due to methodological differences.^34,37^ A recent study using the HSE06 hybrid functional, known for its high accuracy, reported a MnBr_2_ band gap of 4.21 eV.^33^ A comparison between the PBE and HSE06 band structures reveals a similar trend, further validating the present results.

This similarity between both structures is due to the electronic interactions within the monolayer being more intense than the interactions between adjacent monolayers, which are of the van der Waals type. This fact occurs due to the large distance between adjacent monolayers, which is close to 6.48 Å. Thus, the electronic interactions of the system occur predominantly within the monolayer as suggested in other similar studies.^38^ The analysis confirms that bulk and monolayer MnBr_2_ exhibit magnetic semiconductor behavior due to the unpaired spin channels and a sizable band gap.

### 3.3 Projected Density of States (PDOS) Analysis

**Figure 5:**
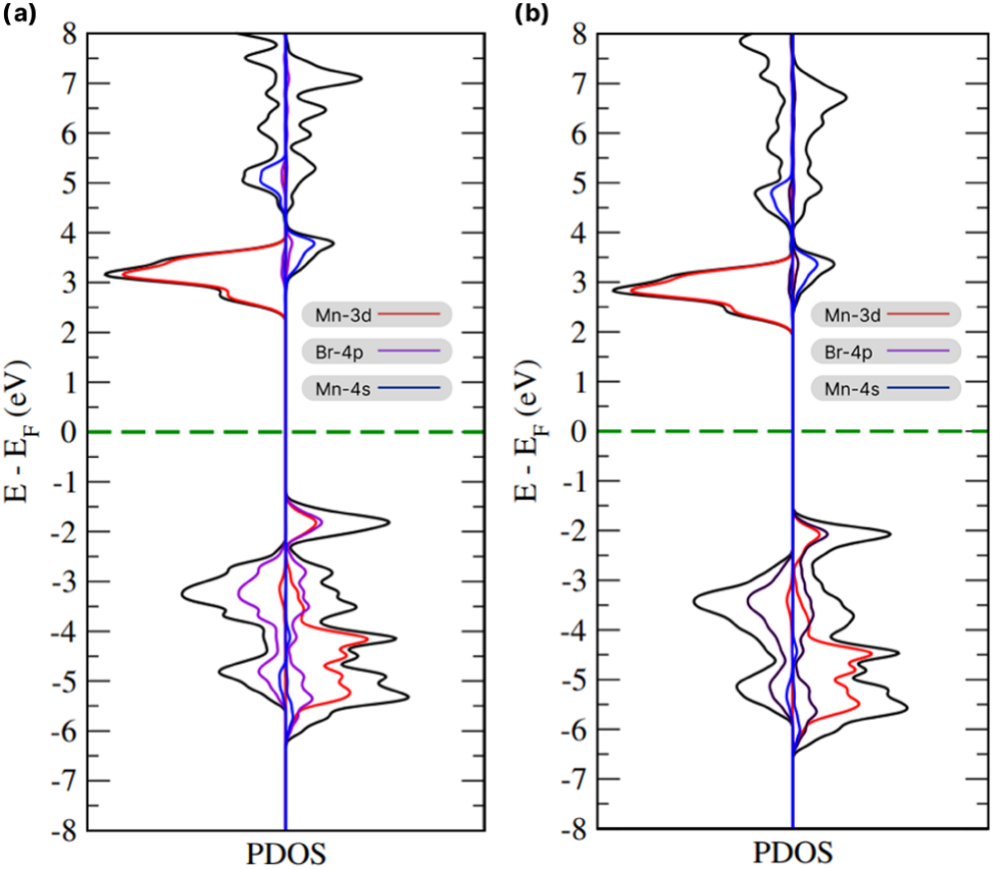
Spin-up and spin-down PDOS plotted on the *x*-axis for (a) bulk and (b) monolayer MnBr_2_. The green dotted lines indicate the Fermi level (*E*_F_) and the black lines represent the total densities of states.

**Figure 6:**
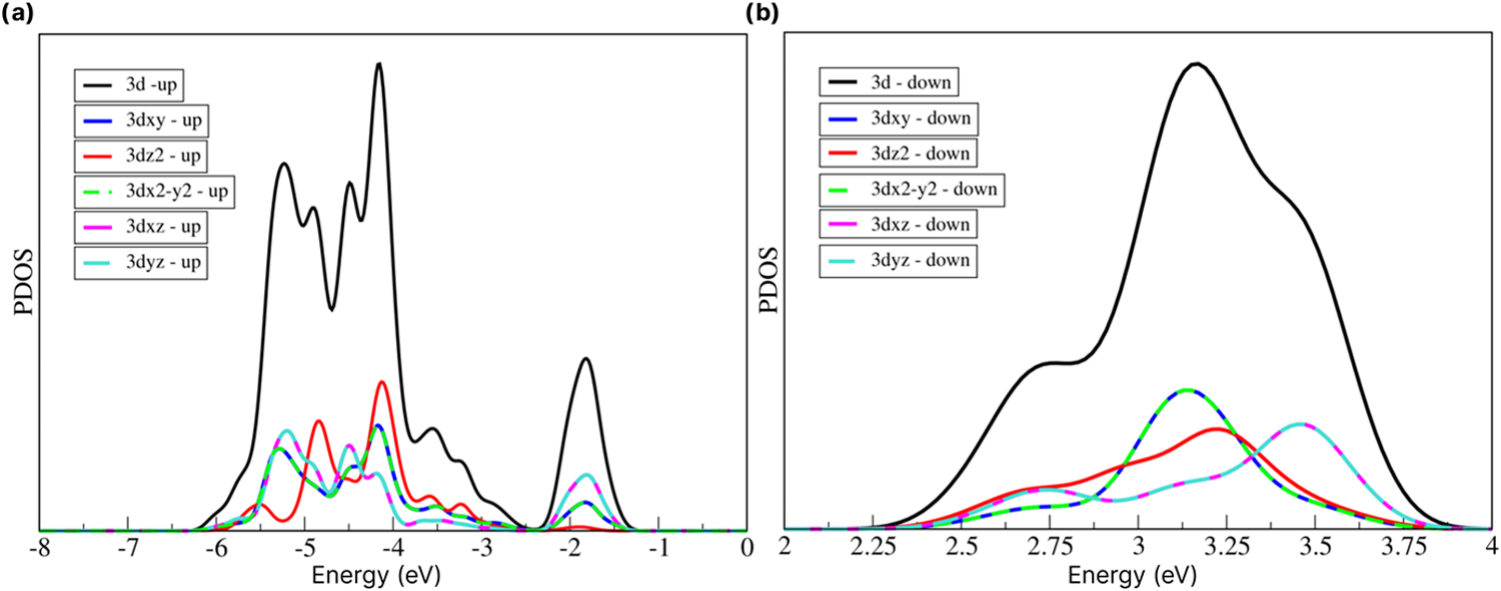
(a) Splitting of the d orbital of the Mn atom with up polarization and (b) with down polarization.

The PDOS for MnBr_2_ was calculated for both the bulk and monolayer forms (Figure 5), further supporting the band structure results and revealing notable similarities. The primary contributions to both spin channels come from the Mn-3d orbitals (red curve) and the Br-4p orbitals (purple curve), indicating d-p orbital hybridization. According to electronic configuration rules, five electrons occupy the Mn-3d orbital. This incomplete filling results in nonzero intrinsic angular momentum, which gives rise to the material’s magnetism, a phenomenon similarly observed in Heusler alloys^39,40^ and TMD-type materials.^41−43^ To detail the contribution of the d orbital of the Mn atom to the system, the PDOS of the orbital separation of the 3d monolayer was calculated as shown in Figure 6.

**Figure 7:**
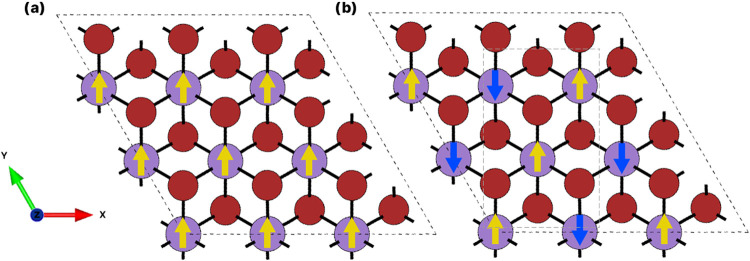
Ferromagnetic (a) and antiferromagnetic ordering (b) for the 3 × 3 × 1 monolayer and bulk forms of MnBr_2_.

**Figure 8:**
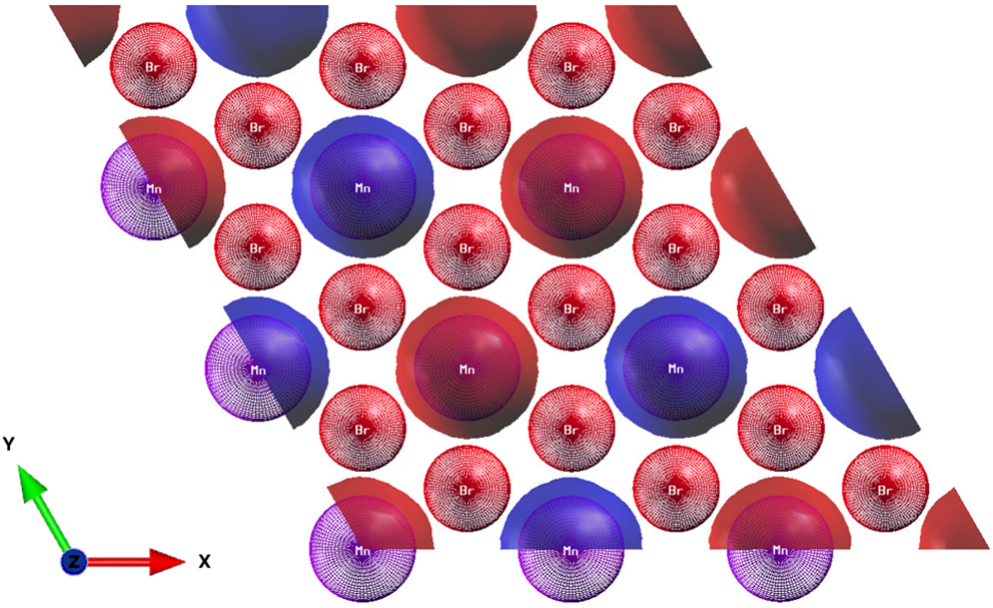
Spin density isosurface with a 0.01 isovalue for the 3 × 3 × 1MnBr_2_ monolayer, where the blue isosurfaces represent Mn atoms with spin-down polarization and the red isosurfaces represent Mn atoms with spin-up polarization.

To further investigate the origin of intrinsic magnetism in MnBr_2_, the magnetic moments for each atom were computed using Mulliken Charge analysis. The spin-polarized calculations indicate that in the bulk form, the Mn atom exhibits a magnetic moment of 5.0 μ_B_, while the Br atom shows zero magnetism. Identical values were found for the magnetic moments of the Mn and Br atoms in the monolayer form, classifying Br as nonmagnetic and confirming the Mn as the primary contributor to the intrinsic magnetism of the material, aligning with Hund’s rule. With this understanding of the magnetic properties of MnBr_2_, the preferred magnetic ordering was then investigated. Figure 7 illustrates the ferromagnetic (FM) and antiferromagnetic (AFM) ordering for the 3 × 3 × 1 monolayer, which consists of nine Mn atoms and 18 Br atoms. To visualize this magnetic ordering, spin density isosurfaces are shown in Figure 8.

### 3.4 Exchange Interaction in the Heisenberg Model

To understand this different magnetic ordering, based on the Heisenberg model, the isotropic exchange interactions (*J*) for each spin configuration were calculated from the energy difference between the AFM and FM states, as follows^44^
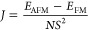
1

**Figure 9:**
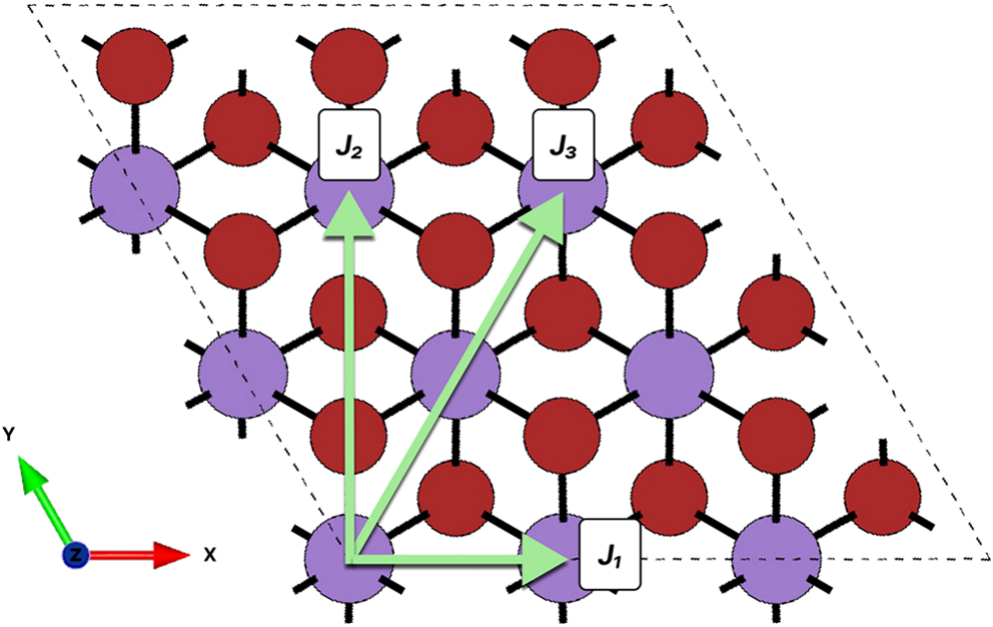
Representation of the exchange interactions between the first three neighbors of Mn in a 3 × 3 × 1 monolayer of MnBr_2_. *J*_1_ corresponds to the nearest neighbor interaction, *J*_2_ corresponds to the next nearest neighbor, and *J*_3_ corresponds to the third nearest neighbor.

*N* denotes the number of magnetic atoms in the unit cell (totaling nine Mn atoms) and *S* is the spin of Mn. Both the monolayer and bulk forms of MnBr_2_ exhibited a more negative total energy for the AFM configurations, confirming the material’s magnetic nature as previously observed in both theoretical and experimental studies.^34,45^ Additionally, the TB2J computational tool was utilized to compute the exchange parameters, as shown in Figure 9, which illustrates the interactions between nearest neighbors within the unit cell, where *J*_1_ corresponds to the nearest neighbor (NN) interaction, *J*_2_ corresponds to the next nearest neighbor (NNN), and *J*_3_ corresponds to the third nearest neighbor (3NN). The *J*_1_ interaction (direct exchange) involves a direct overlap of the wave functions of the two Mn atoms as shown in Figure 9. In contrast, the *J*_2_ interaction (superexchange) includes overlap with the neighboring Br atoms before reaching the next Mn atom. Finally, *J*_3_ describes the interaction among the Mn atoms.

**Figure 10:**
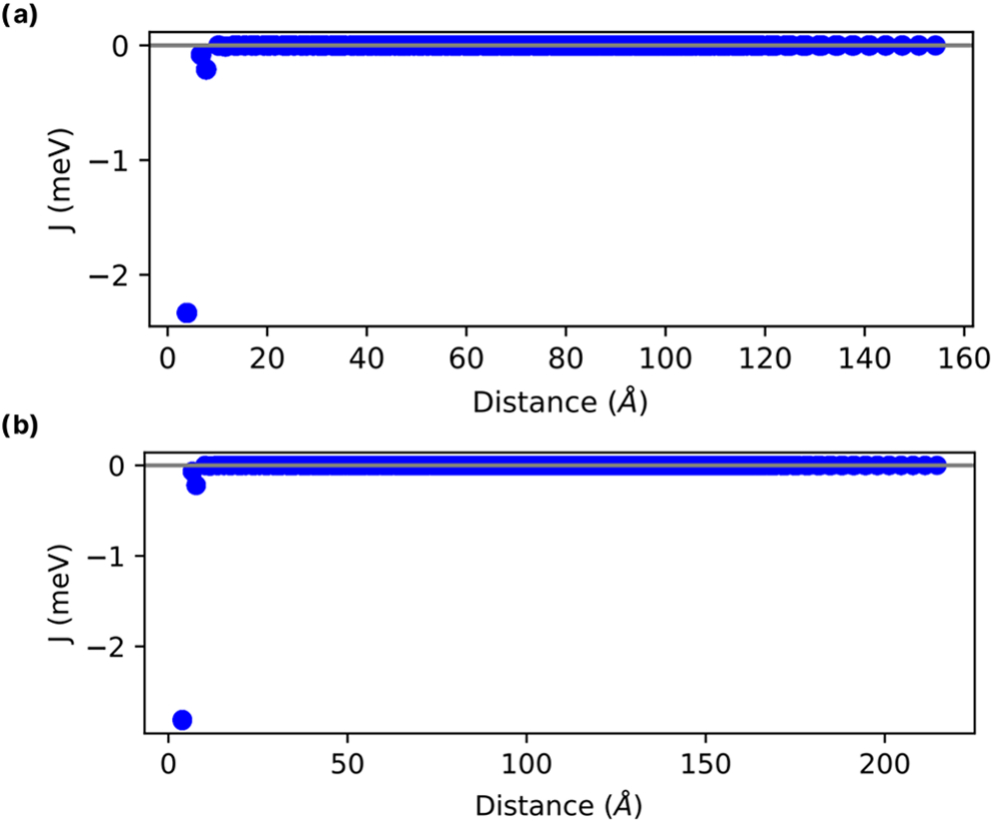
Isotropic exchange interactions (*J*) as a function of distance for the (a) 3 × 3 × 1 bulk and (b) 3 × 3 × 1 monolayer forms of MnBr_2_.

The exchange interactions typically decrease with increasing distance between atoms. Therefore, most interactions become negligible at larger distances within the supercell, limiting effective interactions to nearest neighbors. Results for the monolayer and bulk forms confirm this behavior (Figure 10), as calculated via eq 1.

These exchange parameters indicate the strength of the interactions between pairs of atoms. For positive values of *J*_*i*_, the interaction is classified as ferromagnetic, while negative values indicate an antiferromagnetic interaction. Using the TB2J tool, these parameters were calculated utilizing the Magnetic Force Theorem method. The obtained values are displayed in Table 1.

**Table 1:** Isotropic (In-Plane) Exchange Parameters Obtained via TB2J (*J*_1_, *J*_2_, and *J*_3_) and eq 1 (for *J*), for MnBr_2_ Bulk and Monolayer Forms

MnBr_2_	*J*_1_ (meV)	*J*_2_ (meV)	*J*_3_ (meV)	J (meV)
3 × 3 × 1 monolayer	–0.45	–0.01	–0.035	–2.70
3 × 3 × 1 bulk	–0.38	–0.009	–0.032	–2.52

The patterns obtained for the bulk and monolayer forms were very similar. All the *J*_3_ (3NN) interactions considered in the calculations were antiferromagnetic. The total energy difference between the AFM and FM configurations, Δ*E*_AFM-FM_, for the 3 × 3 × 1 monolayer and bulk forms were −0.152 and −0.142 eV, respectively. To validate these determined values, and knowing that the Mn spin is , the exchange interaction of the material was calculated via the energy difference for each spin configuration, as described by eq 1.

### 3.5 Magnetic Anisotropy Energy

To understand the preferential orientation of magnetism in the material, the magnetic anisotropy energy (MAE) was computed from the total energy of the supercell for the bulk and monolayer forms of MnBr_2_, with spins oriented along two preferential axes (*x* and *z*). Since magnetization in two dimensions is susceptible to spin rotation, the MAE is a critical property, which can be calculated using the following equation
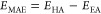
2where *E*_HA_ is the energy of the hard axis (100) and *E*_EA_ is the energy of the easy axis (001). The computed MAE values (*E*_MAE_) for both 3 × 3 × 1 monolayer and bulk MnBr_2_ are presented in Table 2.

**Table 2:** Calculated Magnetic Anisotropy Energy (*E*_MAE_) for MnBr_2_ Bulk and Monolayer Forms

MnBr_2_	*E*_MAE_ (μeV)
3 × 3 × 1 monolayer	61
3 × 3 × 1 bulk	106

The results suggest that the system is more stable when the magnetic moments of the Mn atoms are aligned along the *z*-axis in both the bulk and monolayer supercells. To further investigate how the total energy of the system is influenced by the rotation of the magnetic moment of each Mn atom, the energy difference between the *z*- and *x*-axes was calculated.

**Figure 11:**
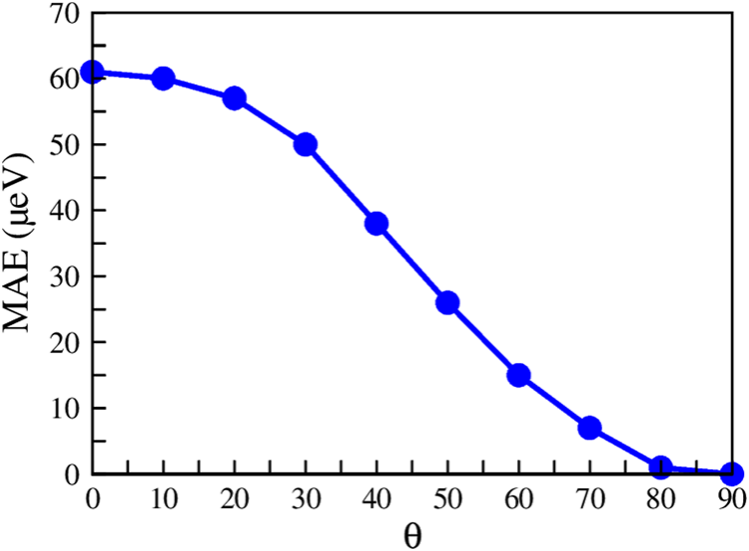
Magnetic anisotropy energy (MAE) as a function of θ angle (angle between the spin magnetic moment and the *z*-axis). The θ angle varied between 0 and 90°, while the azimuthal angle ϕ remained fixed at 0° (*x*-axis).

Using spherical coordinates (θ and ϕ), this calculation was performed by varying the θ angle (the angle between the spin magnetic moment and the *z*-axis) from 0 to 90° (with a step of 10°), while keeping the azimuthal angle ϕ fixed at 0 (*x*-axis). Figure 11 shows the behavior of *E*_MAE_ as a function of θ, demonstrating that the system’s stability decreases and reaches a minimum as the total alignment of the spins shifts toward the *x*-axis.

The MAE is also a significant parameter for evaluating the materials’ thermal stability and suitability for spintronics applications, such as data storage.^46^ The higher the MAE, the more energy is required for the spin to rotate, which consequently increases the thermal stability of the system.

The calculated MAE values for the monolayer and bulk forms of MnBr_2_ were 6.8 μeV/Mn and 11.7 μeV/Mn, respectively, which are significantly higher than those for bulk Fe (1.4 μeV/Fe) and Ni (2.7 μeV/Ni),^47^ two widely used materials in spintronics applications. This suggests that MnBr_2_ could offer enhanced thermal stability for spintronic devices.

## 4 Conclusions

This work investigates the electronic and magnetic properties of manganese bromide (MnBr_2_) in both bulk and monolayer forms. The obtained band structure and the projected density of states indicate that this material exhibits magnetic semiconductor behavior. Additionally, the cleavage energy calculation for the bulk form suggests that transitioning to the monolayer form can be achieved experimentally through mechanical microexfoliation, as demonstrated with graphene.

Analysis of the material’s magnetic ordering reveals that the antiferromagnetic configuration is the most stable. Furthermore, the calculated isotropic exchange interactions for two-dimensional and three-dimensional forms exhibit antiferromagnetic direct exchange and superexchange interactions, respectively.

Calculations of magnetic anisotropy energy demonstrate that the system is more stable when the magnetic moments of the manganese atoms are aligned along the *z*-axis in both the bulk and monolayer supercells. Moreover, significant magnetic anisotropy energy values of 6.8 and 11.7 μeV/Mn, respectively, were obtained for the monolayer and bulk forms, indicating a high degree of thermal stability of the MnBr_2_ material. Ultimately, the results of this study position MnBr_2_ as a promising candidate for technological applications in nanoelectronics and spintronics, where stability and magnetic properties are essential.
